# Microbiology of Urinary Tract Infections in Gaborone, Botswana

**DOI:** 10.1371/journal.pone.0057776

**Published:** 2013-03-04

**Authors:** Andrew J. Renuart, David M. Goldfarb, Margaret Mokomane, Ephraim O. Tawanana, Mohan Narasimhamurthy, Andrew P. Steenhoff, Jonathan A. Silverman

**Affiliations:** 1 Botswana-UPenn Partnership, Gaborone, Botswana; 2 Perelman School of Medicine, University of Pennsylvania, Philadelphia, Pennsylvania, United States of America; 3 McMaster University, Hamilton, Ontario, Canada; 4 Department of Paediatrics and Adolescent Medicine, School of Medicine, University of Botswana, Gaborone, Botswana; 5 National Health Laboratory, Gaborone, Botswana; 6 Microbiology Laboratory, Princess Marina Hospital, Gaborone, Botswana; 7 Bokamoso Private Hospital, Gaborone, Botswana; 8 Children’s Hospital of Philadelphia, Philadelphia, Pennsylvania, United States of America; 9 Department of Pediatrics, University of Washington School of Medicine, Seattle, Washington, United States of America; Northwestern University, United States of America

## Abstract

**Objective:**

The microbiology and epidemiology of UTI pathogens are largely unknown in Botswana, a high prevalence HIV setting. Using laboratory data from the largest referral hospital and a private hospital, we describe the major pathogens causing UTI and their antimicrobial resistance patterns.

**Methods:**

This retrospective study examined antimicrobial susceptibility data for urine samples collected at Princess Marina Hospital (PMH), Bokamoso Private Hospital (BPH), or one of their affiliated outpatient clinics. A urine sample was included in our dataset if it demonstrated pure growth of a single organism and accompanying antimicrobial susceptibility and subject demographic data were available.

**Results:**

A total of 744 samples were included. Greater than 10% resistance was observed for amoxicillin, co-trimoxazole, amoxicillin-clavulanate, and ciprofloxacin. Resistance of *E. coli* isolates to ampicillin and co-trimoxazole was greater than 60% in all settings. HIV status did not significantly impact the microbiology of UTIs, but did impact antimicrobial resistance to co-trimoxazole.

**Conclusions:**

Data suggests that antimicrobial resistance has already emerged to most oral antibiotics, making empiric management of outpatient UTIs challenging**.** Ampicillin, co-trimoxazole, and ciprofloxacin should not be used as empiric treatment for UTI in this context. Nitrofurantoin could be used for simple cystitis; aminoglycosides for uncomplicated UTI in inpatients.

## Introduction

Urinary tract infections (UTI) are considered to be the most common bacterial infection, and it is estimated that they affect up to 150 million individuals annually [1], [2]. There are limited data on the true impact of UTIs in the developing world. In the USA, UTIs are responsible for 7 million physician visits and more than 100,000 hospital admissions annually [3]. The total annual direct and indirect cost due to UTIs in the US alone is estimated to be greater than US$ 1.6 billion [3]. Globally, they cause not only a significant amount of morbidity, but also a significant financial burden [2], [4].

The introduction of antimicrobial therapy has led to profound improvements in the management of urinary tract infections; however antimicrobial resistance is a growing problem and a cause of major concern in many countries [4]–[6]. The main causative agents of UTI are Gram-negative bacteria, notably *Escherichia coli*, however other Enterobacteriaceae and *Staphylococcus saprophyticus* are also commonly involved [7], [8]. Over the past several decades, resistance to many of the commonly prescribed UTI antibiotics - ampicillin, co-trimoxazole, nitrofurantoin, and fluoroquinolones - has emerged [9].

Recent clinical guidelines emphasize the importance of knowing local patterns of susceptibility of coliforms to antimicrobial agents [6], [10]. To date, there have been no studies describing the microbiology and epidemiology of pathogens causing urinary tract infection among pediatric and adult populations in Botswana, a medium income sub-Saharan African setting with a relatively well-developed primary health care system and a total population prevalence of HIV of 17.6% [11]. Our primary objective was to describe the major pathogens causing UTI among pediatric and adult populations, as well as the patterns of antimicrobial resistance locally, with the ultimate aim of guiding empiric therapy. Secondary objectives were to compare differences in these patterns within the public and private healthcare sectors, as well as within inpatient and outpatient settings. Additionally, we sought to explore the impact of HIV status on UTI pathogens and antibiotic resistance patterns.

## Methods

### Ethics Statement

Ethical approval was granted by the institutional review boards at the Botswana Ministry of Health, Princess Marina Hospital (PMH), Bokamoso Private Hospital (BPH) and the University of Pennsylvania. These IRBs also granted waivers of consent, as all data was retrospectively collected and deidentified on our data collection sheets to ensure patient confidentiality.

This was a retrospective study of antimicrobial susceptibility data for urine samples collected at PMH, BPH, or one of their affiliated outpatient clinics. PMH has 525 beds and is the main public tertiary-care hospital in Botswana’s capital city, Gaborone, while BPH has 200 beds and is a private primary, secondary and tertiary-care hospital also located in the capital. Affiliated clinics are located throughout the greater Gaborone area and serve a population of around 300,000 people.

For PMH, we reviewed laboratory records of urine specimens submitted for investigations from patients who presented between January 1, 2007 and December 31, 2009. Samples from BPH, which opened on January 11, 2010 were collected between January 11, 2010 and December 31, 2010. For both hospitals, we included urine samples for patients who were admitted to various inpatient wards, as well as patients who had samples collected at affiliated outpatient clinics. All samples were collected as part of routine patient care.

A urine sample was included in our dataset if it demonstrated growth of a single organism with accompanying antimicrobial susceptibility, and subject demographic data were available. For patients with more than one sample, we included only the first positive sample with susceptibility data. Samples were excluded from the dataset if there were duplicate samples with differing sensitivities, if there were duplicate samples listed under multiple years, or if samples grew multiple pathogens.

Urine microscopy, isolation, and identification of organisms were carried out as part of the routine procedures in the microbiology laboratories at PMH and BPH. Antimicrobial susceptibility testing was conducted at PMH via disc diffusion method according to the Clinical and Laboratory Standards Institute (CLSI) guidelines [12]. Additional testing for extended spectrum beta-lactamase (ESBL) production was not available at PMH during the time period included in the study. BPH used the MicroScan WalkAway automated system (Siemens Healthcare Diagnostics), which included screening for Extended-Spectrum Beta-Lactamase (ESBL) production. Confirmatory testing for ESBL production was carried out using disc diffusion according to CLSI guidelines (CLSI) [12].

Additional data collected included patient age, sex, HIV status and clinical setting.

Analysis was performed using standard descriptive statistics and STATA version 11.1 (College Station, TX). Proportions of resistance were compared using Fisher’s exact and chi-squared tests and p<0.05 was deemed significant.

## Results

At PMH, 680 urine culture samples were evaluated for inclusion. Samples were not cultured if urinalysis was normal. Twenty-eight samples were excluded for the following reasons: a) duplicate samples with matching specimen and medical record numbers, but differing sensitivities (sample with most complete sensitivity data was included) *(n = 8)*; b) duplicate samples with matching medical record numbers and specimen numbers, but with multiple years listed (correct year was confirmed via electronic medical records and duplicate was excluded) *(n = 9)*; c) samples that grew multiple pathogens *(n = 52)*. A total of 652 samples were included from PMH, which included all samples that met our inclusion criteria. Of the samples from PMH, 58% were from outpatient clinics.

At BPH, 760 samples were evaluated for inclusion from BPH. We included a total of 92 samples, with the remainder excluded for either no growth of a single organism, or growth of multiple organisms. The low culture positivity rate at BPH vs PMH may be explained by the fact that specimens were often cultured at BPH without a prior microscopy or dip or despite a negative microscopy or dip. Seventy-three percent of eligible samples from BPH were from outpatient clinics.

Urine dipsticks, as well as urine microscopy, culture, and sensitivities, were carried out inconsistently at both institutions. Cultures at PMH were generally performed on samples with >10 white blood cells (WBC) per high power field (hpf) on microscopy; however, some had cultures performed if they were only positive on dipstick testing for blood, leukocyte esterase, or nitrites. Sometimes cultures were performed without prior microscopy or with normal microscopy.

Information on patient demographics is listed in Table 1. There were no significant differences in the age distribution or gender of patients when comparing the private and public sectors. Inpatients were more likely to be male (P = 0.002) and to be less than 18 years old or 65 years and older (P<0.001). There were broadly equivalent percentages of HIV-positive patients in the public and private settings, although patients were more likely to have an unknown or undocumented HIV status at PMH (P<0.001). However, at PMH, almost half (49%) of the patients with known HIV status were HIV-positive compared to just over a quarter (26%) at BPH (P = 0.002).

**Table 1:** Patient Demographics.

	Princess Marina Hospital (n = 652)			Bokamoso Hospital (n = 92)		
	Inpatient	Outpatient	*Total*	Inpatient	Outpatient	*Total*
**Age** (yrs) Median(IQR)	37 (26–56)	28 (22–41)	*32 (23–49)*	50 (36–62)	36 (29–47)	*37 (30–55)*
<18 yrs	13%	9%	*10%*	4%	12%	*10%*
18–65 yrs	70%	85%	*78%*	80%	76%	*77%*
>65 yrs	17%	7%	*11%*	16%	12%	*13%*
**Gender**						
Female	57%	68%	*63%*	72%	70%	*71%*
Unknown	1%	3%	*2%*	0%	0%	*0%*
**HIV Status**						
Negative	25%	5%	*13%*	36%	42%	*40%*
Positive	20%	8%	*13%*	36%	6%	*14%*
Unknown	55%	87%	*74%*	28%	52%	*46%*

Uropathogens from PMH and BPH are listed in Table 2. There were significant differences in the pathogens isolated when comparing private to public settings (P = 0.001) and inpatient to outpatient settings (P = 0.007). *E. coli* was the most common pathogen in all settings and accounted for 63% of isolates at both PMH and BPH. Sixty-six percent of outpatient isolates were *E. coli*, compared to 58% in inpatient settings. At PMH, *Klebsiella* species were responsible for 14% of UTIs followed by *Proteus* species that were responsible for another 5%. *Klebsiella* and *Proteus* species each accounted for 7% of isolates at Bokamoso.

**Table 2:** Uropathogens.

	Princess Marina Hospital			Bokamoso Hospital		
	Inpatient (n = 276)	Outpatient (n = 376)	*Total (n = 652)*	Inpatient (n = 25)	Outpatient (n = 67)	*Total (n = 92)*
***E. coli***	58%	66%	***63%***	52%	67%	***63%***
***Klebsiella spp.***	16%	12%	***14%***	16%	3%	***7%***
***Proteus spp.***	7%	4%	***5%***	4%	7%	***7%***
***Coagulase negative*** ***S. aureus***	4%	4%	***4%***	0	3%	***2%***
***Streptococcus spp.***	1%	4%	***3%***	0	0	***0***
***S. aureus***	2%	3%	***2%***	0	1%	***1%***
***Enterobacter spp.***	3%	2%	***2%***	4%	1%	***2%***
***Enterococcus spp.***	3%	2%	***2%***	4%	4%	***4%***
***Citrobacter spp.***	2%	2%	***2%***	4%	4%	***4%***
***Morganella morganii***	1%	1%	***1%***	0	0	***0***
***Group B Streptococcus***	1%	1%	***1%***	4%	4%	***4%***
***Pseudomonas spp.***	1%	0	***1%***	12%	0	***3%***
***S. saprophyticus***	0	1%	***<1%***	0	3%	***2%***
***Salmonella spp.***	<1%	0	***<1%***	0	0	***0***

At BPH, 10 isolates (11%) were found to produce ESBL. Six were *E. coli* isolates, three were *Proteus* isolates, and one was a *Klebsiella* isolate.

**Figure 1:**
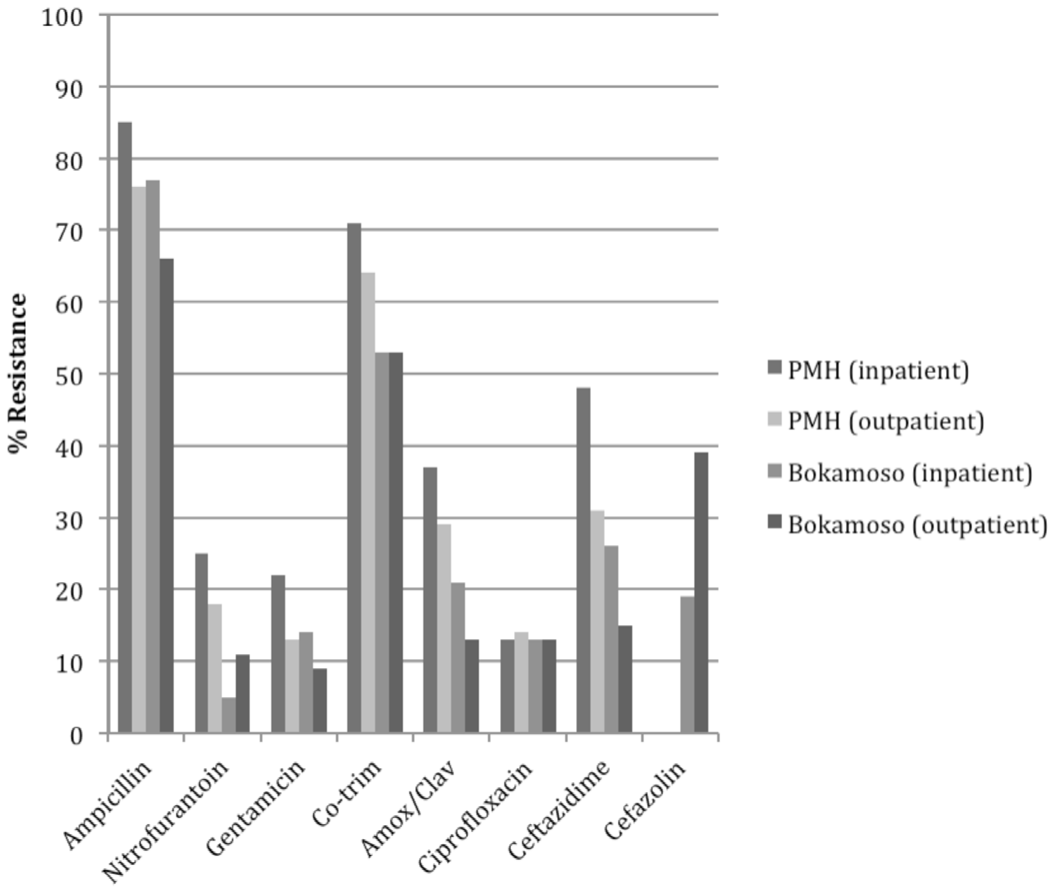
Antimicrobial resistance patterns of all UTI pathogens. * *Less than 15 samples analyzed for these antimicrobials as noted: PMH-Outpatients (ciprofloxacin, ceftazidime).

**Figure 2:**
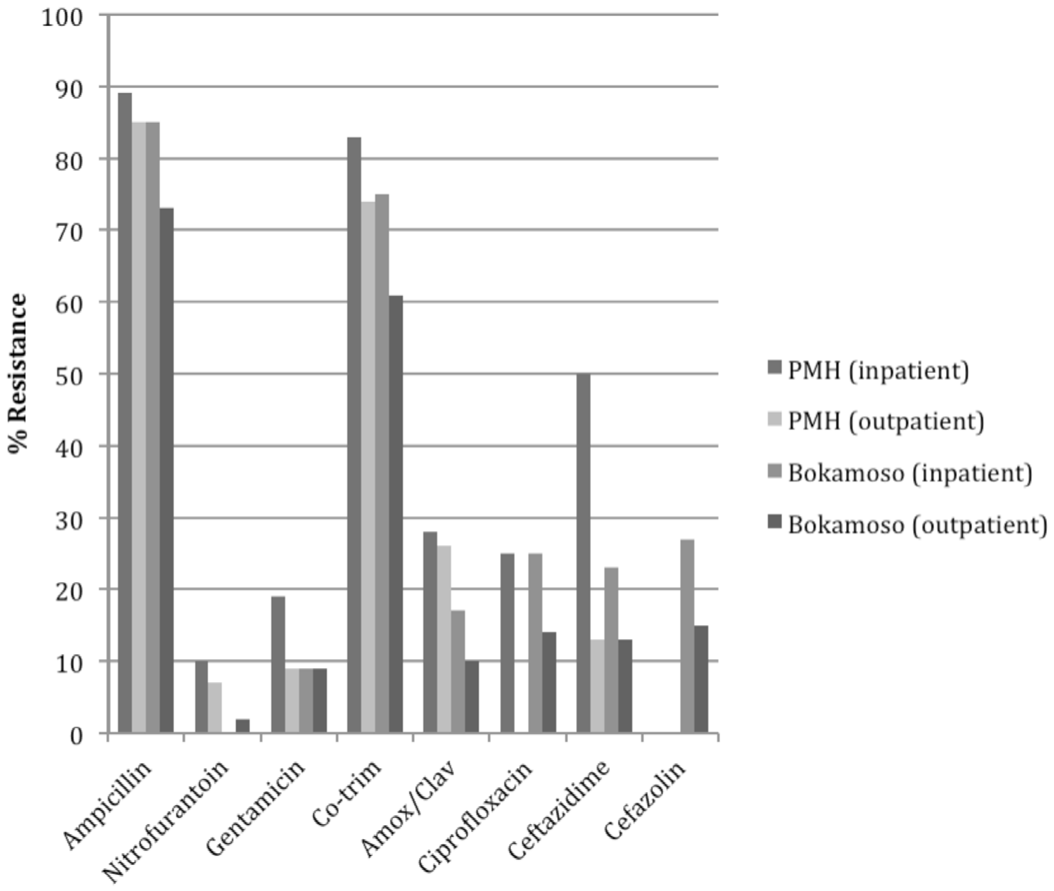
Antimicrobial resistance patterns of E. coli isolates. * *Less than 15 samples analyzed for these antimicrobials as noted: BPH-Inpatients (all antibiotics), PMH-Outpatients (ciprofloxacin, ceftazidime), PMH-Inpatients (ciprofloxacin, ceftazidime).

Figure 1 illustrates overall resistance patterns for all uropathogens stratified by study site. Given that it was the predominant organism, analysis focused on resistance patterns of *E. coli* isolates (Figure 2). Among *E. coli* isolates, there was no significant difference in resistance to ampicillin when comparing samples from in- and outpatient settings. However, resistance was significantly higher among samples from patients at PMH compared to those from BPH (86% vs. 76%; P = 0.034). Resistance to gentamicin was higher among samples from inpatients compared to outpatients (P = 0.015) and the public sector compared to the private sector (P = 0.018). Resistance to co-trimoxazole was also higher among samples from inpatients (P = 0.017) and the public sector (P = 0.034). Nearly 30% of *E. coli* isolates at PMH were resistant to amoxicillin/clavulanate compared with 11% from BPH (P = 0.016). However, another 13% of samples from BPH demonstrated intermediate resistance.

Resistance patterns to nitrofurantoin, ceftazidime, and ciprofloxacin did not differ significantly based on setting (P>0.05). Overall resistance to nitrofurantoin was ten percent or lower in all settings. Twenty-five percent of *E. coli* isolates were resistant to ciprofloxacin in the inpatient settings in both the public and private sectors with somewhat lower resistance in outpatient settings.

Data were not available for resistance patterns of first- or second-generation cephalosporins at PMH. There was no significant difference in resistance patterns for cefazolin or cefuroxime at BPH when comparing inpatient and outpatient settings (P>0.05). There was 18 percent resistance to both cefazolin and cefuroxime among *E. coli* isolates at BPH, with an additional 10 percent and three percent showing intermediate resistance respectively.

Sub-analyses also compared the differences in microbiology and antimicrobial susceptibilities between samples from pediatric (<18 years old) and adult populations. There were no significant differences in the pathogens causing UTI when comparing these populations. However, compared to those from adult patients, *E. coli* isolates from pediatric patients did have higher levels of resistance to gentamicin (23% vs 11%) (P = 0.045) and co-trimoxazole (88% vs 74%) (P = 0.031).

We further explored the association between known HIV status, uropathogens, and antimicrobial resistance of E. coli isolates. HIV status had no significant impact on the uropathogens that caused UTI in any setting. However, HIV status was associated with a difference in the resistance of *E. coli* isolates to co-trimoxazole. *E. coli* isolates from known HIV-positive patients demonstrated significantly higher resistance to co-trimoxazole compared to isolates from known HIV-negative patients (95% vs. 72%) (P<0.001). HIV status did not affect resistance patterns to the other antimicrobials that we examined.

## Discussion

*E. coli* accounted for more than 60% of urinary isolates in both the public and private sectors in this study. *Klebsiella* and *Proteus species* were the next most common pathogens isolated. These data are consistent with findings from work in neighboring South Africa [4], [13].

Resistance of *E. coli* isolates to ampicillin and co-trimoxazole was high across all settings although there were statistically significant differences according to locale. *E. coli* isolates from the public sector demonstrated higher levels of resistance to both antibiotics compared to the private sector. Isolates from inpatients were more likely to demonstrate resistance to co-trimoxazole compared to outpatient samples. This latter difference could largely be explained by HIV status and it is highly likely in a Botswana setting that inpatients have a higher HIV prevalence than outpatients. *E. coli* isolates from HIV-positive patients demonstrated higher resistance to co-trimoxazole compared to those samples from patients known to be HIV-negative. This is likely due to the common use of co-trimoxazole for the treatment of and prophylaxis against *Pneumocystis jirovecii* pneumonia among HIV-positive patients.

Recent work in Nigeria has explored the association between HIV status and UTI [14]. Omoregie and Eghafona demonstrated that known HIV-positive patients on HAART had a higher prevalence of asymptomatic UTI compared to HIV-negative patients, although there was no association between CD4+ count and asymptomatic UTI [14]. Our work demonstrates that in the greater Gaborone region, HIV status does not affect the types of pathogens causing UTI. Furthermore, HIV status was not associated with any differences in antibiotic resistance levels, except in the case of co-trimoxazole.

There were a number of limitations in our study that are intrinsic to a study with a retrospective design. Specifically, the diagnosis of UTI would have benefitted from consistent recording of a urine dipsticks, as well as urine microscopy, culture, and sensitivities. However, these were carried out inconsistently at both institutions and represent an opportunity to improve overall care. Additionally, there were inconsistencies in record keeping at PMH. Laboratory data were intermittently recorded in either paper logbooks or in electronic databases - therefore there are some missing data from our analyses that might have impacted our results. Missing data may also have affected our analysis of HIV status. Almost three-quarters of patients at PMH and nearly half of patients at BPH had an undocumented HIV status at the time their urine samples were sent and these samples could not be included in the HIV analysis. These limitations would be adequately addressed through improved prospective UTI surveillance strategies at both sites.

### Conclusions

The epidemiology of UTIs in Botswana appears in general to be similar to that found in much of the world. HIV status did not seem to significantly impact the microbiology of UTIs in our context but did impact antimicrobial resistance to co-trimoxazole. Findings from this retrospective study suggest that antimicrobial resistance has already emerged to most oral agents. Greater than 10% resistance was observed for amoxicillin, co-trimoxazole, amoxicillin/clavulanate, and ciprofloxacin. This makes empiric management of outpatient UTIs challenging. Resistance of *E. coli* isolates to ampicillin and co-trimoxazole was greater than 60% in all settings examined in our study. These agents should not be used as empiric treatment for UTI in the context of Botswana. Additionally, given that there was greater than 20% resistance to ciprofloxacin among inpatient *E. coli* isolates, its use would otherwise be inappropriate as an empiric agent in this setting.

Overall resistance to nitrofurantoin was low in all settings, suggesting that it could be used as empiric monotherapy for simple cystitis. Aminoglycosides are an appropriate alternative in the inpatient setting when there is not a concern regarding nephrotoxicity and when enteral therapy is not an option and bacteremia is not suspected.

A standardized approach to the diagnostic workup of patients with suspected UTI in these settings would likely improve overall care. In addition, improved record keeping and a prospective surveillance system are needed in these Botswana hospitals in order to facilitate regular surveillance of antibiotic resistance as these levels continue to change.
